# Lee Silverman Voice Treatment versus standard speech and language therapy versus control in Parkinson’s disease: preliminary cost-consequence analysis of the PD COMM pilot randomised controlled trial

**DOI:** 10.1186/s40814-021-00888-y

**Published:** 2021-08-09

**Authors:** Sarah Scobie, Sue Jowett, Tosin Lambe, Smitaa Patel, Rebecca Woolley, Natalie Ives, Caroline Rick, Christina Smith, Marion C Brady, Carl Clarke, Cath Sackley

**Affiliations:** 1 Nuffield, Trust, London, UK; 2 Health Economics Unit, Institute for Applied Health Research, University of Birmingham, Birmingham, UK; 3 Institute of Population Health Sciences, University of Liverpool, Liverpool, UK; 4 Birmingham Clinical Trials Unit, Institute for Applied Health Research, University of Birmingham, Birmingham, UK; 5 Nottingham Clinical Trials Unit, University of Nottingham, Nottingham, UK; 6 Psychology and Language Science, University College London, London, UK; 7 Nursing, Midwifery and Allied Health Professions Research Unit, Glasgow Caledonian University, Glasgow, UK; 8 Department of Neurology, Sandwell and West Birmingham Hospitals NHS Trust, Birmingham, UK; 9 Faculty of Life Sciences and Medicine, King’s College London, London, UK

**Keywords:** Parkinson’s disease, Pilot randomised controlled trial, Speech and language therapy, Cost-consequence analysis

## Abstract

**Background:**

The PD COMM pilot randomised controlled trial compared Lee Silverman Voice Treatment (LSVT® LOUD) with standard NHS speech and language therapy (SLT) and a control arm in people with Parkinson’s disease (PwPD) with self-reported problems with voice or speech. This analysis compares costs and quality of life outcomes between the trial arms, and considers the validity of the alternative outcome measures for economic evaluations.

**Methods:**

A comparison of costs and outcomes was undertaken alongside the PD COMM pilot trial involving three arms: LSVT® LOUD treatment (*n* = 30); standard NHS SLT (*n* = 30); and a control arm (*n* = 29) excluded from receiving therapy for at least 6 months after randomisation unless deemed medically necessary. For all trial arms, resource use and NHS, social care and patient costs and quality of life were collected prospectively at baseline, 3, 6, and 12 months. Total economic costs and outcomes (EQ-5D-3L, ICECAP-O) were considered over the 12-month follow-up period from an NHS payer perspective. Quality of life measures for economic evaluation of SLT for people with Parkinson’s disease were compared.

**Results:**

Whilst there was no difference between arms in voice or quality of life outcomes at 12 months, there were indications of differences at 3 months in favour of SLT, which need to be confirmed in the main trial. The estimated mean cost of NHS care was £3288 per patient per year for the LSVT® LOUD arm, £2033 for NHS SLT, and £1788 for the control arm. EQ-5D-3L was more strongly correlated to voice impairment than ICECAP-O, and was sensitive to differences in voice impairment between arms.

**Conclusions:**

The pilot did not identify an effect of SLT on disease-specific or economic outcomes for PwPD at 12 months; however, there appeared to be improvements at 3 months. In addition to the sample size not powered to detect difference in cost-consequence analysis, many patients in the control arm started SLT during the 12-month period used for economic analysis, in line with the study protocol. The LSVT® LOUD intervention was more intense and therefore more costly. Early indications suggest that the preferred economic outcome measure for the full trial is EQ-5D-3L; however, the ICECAP-O should still be included to capture a broader measure of wellbeing.

**Trial registration:**

International Standard Randomised Controlled Trial Number Register: ISRCTN75223808. Registered 22 March 2012.

**Supplementary Information:**

The online version contains supplementary material available at 10.1186/s40814-021-00888-y.

## Key messages regarding feasibility

- There is a lack of published evidence on costs and cost-effectiveness of Speech and Language Therapy (SLT) in Parkinson’s disease and uncertainties regarding economic outcome measures.
- Resource use data collection is feasible in this patient group, and differences in cost were mainly due to differences in SLT costs. The economic evaluation for the main trial should also consider carer and patient-incurred costs.
- The Euro-Qol EQ-5D-3L was both valid and sensitive when considering voice impairment levels and changes in levels. The analysis shows that for the main trial, the most appropriate economic measure is EQ-5D-3L; however, ICECAP-O can also be used.

## Background

Parkinson’s disease (PD) is a progressive neurological disease, which is estimated to affect 100–180 people per 100,000 population [1]. PD is primarily a movement disorder, not only resulting in disability and impaired quality of life (QoL), but can also result in anxiety and depression, and impaired cognition [2]. The presentation of symptoms varies between individuals and through the course of the disease [2]*.*

An estimated 70% of People with Parkinson’s disease (PwPD) in developed countries have a speech disorder [3], with the occurrence and severity of speech disorders increasing with severity of PD [4]. Among PwPD, 38% report speech among their top 4 concerns [3] and three-quarters report that their speech has deteriorated. PwPD’s perception of their own speech is not easily measured using self-assessed quality of life tools [5], and PwPD rate their speech as worse than their communication partners’ [6, 7].

Lee Silverman Voice Treatment (LSVT) is an established SLT approach developed for PwPD, and works by encouraging participants to pay attention to speech outputs [8]. LSVT® LOUD is a commercially patented programme comprising of 16 60-min sessions of SLT delivered over 4 weeks [9]. An audit of Parkinson’s Disease services within the UK National Health Service (NHS), estimated that 80% of service providers offer a version of the intervention [10], although only 35% of services offer LSVT® LOUD to all patients assessed as requiring SLT for communication problems. The standard NHS SLT treatment is typically 6–8 weekly sessions, administered as per local practice by state-registered speech and language therapists and expected to typically involve one session of 45 min per week for 6–8 weeks of varying content as determined by patient need [11]. Systematic reviews [12, 13] have concluded that there is currently insufficient evidence on the clinical and cost effectiveness of LSVT® LOUD compared with standard SLT or no treatment. No studies have been identified which estimate the cost of SLT in PwPD. The PD COMM trial which is ongoing will address this gap by comparing the clinical effectiveness and cost-effectiveness between LSVT® LOUD, NHS SLT, and a control arm [14]. The PD COMM pilot trial was undertaken to assess the feasibility and acceptability of a large-scale RCT [14].

The objectives of this economic analysis were to test data collection methods, compare cost and outcome data for the alternative interventions in the PD COMM pilot trial, and to consider the validity of alternative outcome measures for economic evaluation in the full trial.

## Methods

### Pilot trial

The methods and results of the pilot trial are reported in detail elsewhere [11, 14]. In brief, a randomised controlled trial (RCT) design was used in which participants were randomised to receive one of three alternative treatments:

- LSVT® LOUD: Lee Silverman Voice Treatment
- NHS SLT: NHS speech and language therapy
- Control: No intervention in first 6 months, unless deemed medically necessary

People with idiopathic PD and self-reported problems with voice or speech who had not received SLT for PD speech-related problems in the last 2 years and who did not have dementia were approached in their normal outpatient appointment. Following consent, participants completed a baseline assessment prior to randomisation. Participants and therapists were informed of the treatment allocation, but assessors of the vocal assessments were blind to treatment allocation. Both NHS SLT and LSVT® LOUD were completed in community-based settings (in some cases in the patient’s home) or in outpatient neurology units. Information on resource use and outcomes was collected at baseline, and then at 3, 6, and 12 months post-randomisation.

### Economic analysis

A cost consequence analysis (CCA) was undertaken, analysing and presenting costs and outcomes separately [15]. This is in line with recommendations for the analysis of pilot studies [16]. The costs and consequences were compared at 12 months for: LSVT® LOUD versus NHS SLT; NHS SLT versus control; and LSVT® LOUD versus control. The focus of the primary cost analysis was from an NHS perspective. Additional information on social care costs, private health care usage, and out of pocket expenditure was reported separately, but not as part of overall costs due to limited data. In line with the main analysis of the pilot study, a complete case analysis was undertaken [14]. Cases were excluded where there was missing resource use data at one or more time points. All analyses were undertaken using STATA 16.1.

### Resource use

A micro-approach to costing was used covering: trial data on SLT delivered to LSVT® LOUD and NHS patients; estimates of set-up costs for LSVT® LOUD; and other NHS, residential, social services, and direct patient costs collected from a specifically designed resource use questionnaire completed by participants. Primary, community and outpatient health services were captured: information on inpatient care was not collected, due to limited relevance to the intervention. Medication was recorded at baseline and was assumed to be unchanged over the 12-month period. Whilst there may be small medication changes over 12 months, these are expected only to have a small effect on costs, and SLT interventions are expected to have minimal, if any, impact on medication. Resource use which was recorded by patients in the “other” subsections on the form was reallocated where possible to the relevant existing resource use categories. Except for GP and practice nurse visits, the location of NHS services was not recorded, and so other services were costed on the basis that they took place on NHS premises. Resource use was summarised for each arm and reported separately for privately funded services.

To ensure that any visits for SLT in addition to the trial were captured, visits for SLT were recorded on both the resource usage questionnaire by participants and as part of the session logs by speech and language therapists. These could be visits by patients in the control arm who may have started SLT after 6 months, in line with the protocol, or further SLT after the end of the intervention by the intervention arms. Some participants also reported SLT visits as outpatient appointments. Resource use which double counted activity (between participants and therapist reported data, and between hospital and community visits reported by participants) was excluded. Trial sessions were costed based on recorded length of sessions. The mean session length for LSVT was 62 min (range 44–90 min) and 54 min for NHS SLT (range 36–84 min). Non-trial additional visits were costed based on an average estimated session length of 60 min. Location was recorded for trial sessions: speech and language therapists’ travel time for home visits was estimated to be 1 h per visit.

LSVT® LOUD requires the speech and language therapists to attend a specialised 2-day training course. The cost of training per participants treated was estimated using national data on SLT service delivery on the basis of cost of training per patient in a typical SLT (Supplementary Table 1).

### Unit costs of health and social care

Costs of care were estimated using national costing data [17] where available, or other appropriate sources [18] (Supplementary Table 2). The pricing year was 2014/15. Unit costs were multiplied by resource use to calculate costs for each trial arm. Medication costs were estimated from the British National Formulary [19–21] based on dose, quantity and formulation for PD-related drugs the participant was receiving at baseline.

### Patient costs

Direct care costs were estimated for private services, using published data from relevant care providers [22] (Supplementary Table 2). Out of pocket travel and other costs reported by participants in the resource use questionnaire were summarised. Information on the impact of PD on participants and carers’ work and usual activities was not collected in detail, and there was no information about previous work, so it was not possible to estimate productivity loss. Although patients may also contribute to social care costs, this split of costs was not available.

### Outcomes

A number of validated outcome measures were used in the pilot [11, 14]. This analysis focuses on the economic measures EuroQoL EQ-5D-3L [23] and ICECAP-O [24], the summary index and communication domain score for the Parkinson’s Disease Questionnaire-39 (PDQ-39) QoL measure [25], and the voice measure Voice Handicap Index (VHI) [26]. EQ-5D-3L includes five questions addressing health-related QoL: mobility, self-care, usual activities, pain/discomfort, anxiety/depression [23], and was valued using UK weights [27]. ICECAP-O includes five questions covering the capability attributes: attachment, security, role, enjoyment, control [24] and was valued using weights generated using best–worst scaling [28]. Differences between arms in the change in outcome measures between baseline and 3, 6, and 12 months were estimated using a linear regression model, adjusting for the baseline value of the outcome. Quality adjusted life year (QALY) differences at 12 months were calculated using the area under the curve method using QoL values at baseline, 3, 6, and 12 months, and adjusting for baseline EQ-5D-3L score and duration of disease [29]. Participants with missing outcome data were excluded and a complete case analysis was undertaken.

### Analysis

Detailed analysis of costs was undertaken only for NHS costs, due to limited data collection and information on social care, private, and direct patient costs. NHS costs (with and without SLT) and SLT costs for each arm were estimated; bias-corrected bootstrapping (5000 replications) was undertaken to produce 95% confidence intervals, allowing for potential skewness in the data [30]. Where there was a notable cost outlier, sensitivity analysis was undertaken recalculating costs after removing that observation. The influence of baseline variables on cost was examined through a regression model, in order to robustly estimate the difference in cost between arms. Based on findings from economic burden studies of PD [31, 32], the variables considered for the linear regression model were duration of PD, baseline Parkinson’s-related QoL (PDQ-39 summary index), and Hoehn and Yahr (H&Y) severity score. Adjusted *R*^2^ values were compared to determine which variables were the strongest contributors to the explanatory value of the model. A second model was also tested including baseline VHI, PDQ-39 communication domain score and EQ-5D-3L utility at baseline. The difference between interventions was then estimated using bias-corrected bootstrapping to generate 95% confidence intervals.

### Validity analysis

An exploratory analysis of the validity of EQ-5D-3L and ICECAP-O for speech impairment in PD was also undertaken. Convergent validity of the EQ-5D-3L and ICECAP-O utility measures for PD and speech-related measures was assessed, using correlation analysis, to see if the measures converge with other measures as expected [33]. The analysis was conducted at baseline for all the participants in the pilot study to maximise the data available for analysis. Pearson’s correlation was used for correlations between summary scores, and Spearman’s rank for correlation between responses to individual questions. A correlation coefficient of 0.5 or more was considered strong, between 0.3 and less than 0.5 moderate, and below 0.3 weak [34]. Differences in VHI, PDQ-39, ICECAP-O score, and EQ-5D-3L at baseline were also compared to assess if outcome measures are able to discriminate between groups [33]. The groups used in this comparison were participants with less severe versus more severe PD, defined as a H&Y score of two or below versus 2.5 and above [35]; and participants reporting that speech impacted on their social activities versus other participants. The analyses were undertaken using t-tests with means and 95% confidence intervals presented.

## Results

### Baseline characteristics

Baseline characteristics of participants in each response arm are reported in Table 1: the LSVT® LOUD arm had longer average duration of PD, and were receiving higher therapy doses of medication, but severity of disease was not consistently higher.

**Table 1:** Baseline characteristics by treatment arm

Key characteristics	LSVT	NHS SLT	Deferred
	(*n* = 30)	(*n* = 30)	(*n* = 29)
**Demographic**			
Age, mean (SD)	67 (8.4)	68 (10.3)	65 (7.5)
Gender: *n* (%) male	23 (77)	23 (77)	23 (79)
**Clinical history**			
Years since diagnosed, mean (SD)	6.1 (3.7)	5.6 (4.2)	4.9 (3.9)
Severity (H&Y stage) 2.5 and above, *n* (%)	10 (34)	13 (44)	6 (20)
Number of PD medications, mean	2.1 (1)	1.9 (1)	1.6 (0.9)
**Perception of speech**^a^**: *n* (%)**			
Patient perceives speech/voice problems	23 (82)	23 (79)	
Speech affects social activities	18 (64)	14 (48)	

^a^Collected as part of initial SLT assessment, so only available for LSVT and NHS SLT groups

### Resource use

Use of NHS and social care resources by participants in each arm over 12 months was summarised for participants where resource use data was available at all three time points (Supplementary Table 3). Across all the treatment arms the predominant services accessed by participants were primary care, outpatient services, and other therapists, particularly physiotherapists. Few participants reported receiving social care services. However, two participants were in residential care (social care funded) for the duration of the study. Participants reported similar levels of resource use at 3, 6, and 12 months.

### Resource use related to the intervention

Participants in the LSVT® LOUD arm had over twice as many sessions and SLT time (mean 14.4) as participants in the NHS SLT arm (mean 5.6), and both arms also received additional non-trial sessions (mean sessions 3.6 for LSVT® LOUD and 1.6 for NHS SLT). Participants in the control arm received a mean of 5 SLT sessions over 12 months, with 18 participants reporting receiving SLT in their 12 month resource use questionnaire. LSVT® LOUD patients who completed the first week of treatment (4 sessions) finished the course. Two participants who had four or fewer sessions stopped treatment due to issues related to availability of staff. In the NHS arm, 29 participants had at least one session and the sessions per patient reduced over time; 1 participant received more than 8 sessions.

### Costs

Costs for each type of service were summarised for NHS and social care (Table 2) based on unit costs and resources used (Supplementary Table 2). Medication costs were significant and were higher in the LSVT® LOUD arm, and can be attributed to this arm presenting with more mean PD medications prescribed at baseline (Table 1). Further details of medication costs are presented in Supplementary Table 4. Other major areas of NHS cost were out-patient appointments, GPs and primary and community nursing services. In line with service use, social care contributed little to the cost, with the exception of the two participants in residential care. Participants’ direct costs included travel (43 patients), medication (7 patients), use of private therapists, such as physiotherapists, and expenditure such as installing a stair lift, tilt/riser chairs, and other equipment (16 patients) (Supplementary Tables 5 and 6).

**Table 2:** Mean NHS and social care costs over 12 months, per patient (2014/15 costs)

**Type of resource** Mean (SD)	**LSVT® LOUD** (*n* = 24) £	**NHS SLT** (*n* = 24) £	**Control** (*n* = 28) £
**Speech and language therapist (SLT)**			
Set-up costs	18.6	–	–
SLT trial sessions	940 (340)	343 (232)	–
SLT travel	104 (265)	13 (64)	–
Additional SLT sessions	51 (147)	57 (123)	325 (389)
**Sub-total**	**1113 (496)**	**413 (247)**	**325 (389)**
**Medication**	**883 (691)**	**616 (646)**	**577 (598)**
**Primary care and community nursing services**			
GP surgery	165 (172)	166 (135)	143 (141)
GP home	57 (236)	68 (160)	17 (48)
Practice nurse	56 (54)	42 (59)	43 (58)
Practice nurse home	6 (20)	7 (19)	5 (22)
Parkinson’s disease nurse specialist	106 (87)	70 (76)	111 (58)
Health visitor	15 (34)	4 (15)	4 (14)
**Sub-total**	**405 (357)**	**358 (196)**	**323 (192)**
**Therapists and other healthcare professionals**			
Social worker	16 (52)	10 (35)	0 (0)
Physiotherapist	84 (144)	85 (171)	62 (82)
Occupational therapist	31 (62)	22 (62)	13 (37)
Other including home care	281 (1301)	53 (149)	16 (77)
**Sub-total**	**412 (1289)**	**170 (270)**	**91 (122)**
**Outpatient appointments**			
PD medical appointments	266 (231)	216 (222)	135 (139)
PD other appointments	30 (87)	34 (89)	76 (168)
Other appointments	197 (189)	235 (234)	211 (295)
**Sub-total**	**492 (338)**	**486 (247)**	**422 (435)**
Social care services	26 (98)	137 (614)	0.4 (2)
Residential care (/week)	0 (0)	4810 (16,296)	0 (0)

The mean NHS costs, with and without SLT, are shown in Table 3, with 95% CIs (based on 5000 bootstrapped replications). The SLT costs were similar between the NHS SLT and control arm, but substantially higher for the LSVT® LOUD arm. Excluding SLT, NHS costs were more similar across arms, although still higher for the LSVT® LOUD arm, as a result of higher medication and community service resource use. Removal of an outlying observation from the LSVT® LOUD arm reduced the all-NHS services cost to £3071 (95%CI £2644–3533) and the NHS services excluding SLT costs to £1946 (95%CI £1504–2448), both still higher than the other arms.

**Table 3:** Cost estimate/per patient for NHS services, with and without SLT, and QALYs for each treatment arm (2014/15 costs)

	**£/patient (bootstrapped 95% CI)**			**Unadjusted 12-month QALYs**
	NHS services excl SLT	SLT	All NHS services	Mean (SD)
**LSVT** (***n*** = 24)	2175 (1631, 2875)	1113 (923 to 1310)	3288 (2776 to 3972)	0.56 (0.28)
**NHS SLT** (***n*** = 24)	1620 (1326 to 1978)	413 (332 to 529)	2033 (1728 to 2379)	0.63 (0.21)
**Control** (***n*** = 28)	1413 (1144 to 1726)	325 (195 to 478)	1738 (1450 to 2064)	0.63 (0.22)

Regression analysis indicated that duration of illness was the strongest confounding factor, with an increase of £133 per year since diagnosis of PD, adjusted for all other factors. Adjusting for illness duration, the NHS costs for the LSVT® LOUD arm were £948 higher than the NHS SLT arm and £1345 higher than the control arm (Table 4), similar to the cost of SLT for the LSVT® LOUD arm of £1113 (Table 2). The costs for the NHS SLT arm were £307 higher than the control arm (95% CI £736 to £132). An additional model was also tested including baseline VHI, EQ-5D-3L, and PDQ-39 communication score (Table 4). Duration of illness was the strongest confounding factor for all comparisons; however, this had a much weaker influence on the NHS SLT vs. control comparison.

**Table 4:** Cost differences between treatment arms

	**Mean, £**	**95% CI**
**Unadjusted analysis**		
LSVT vs NHS SLT* (*n* = 48)	1256	(601, 1946)
NHS SLT vs control* (*n* = 52)	294	(− 149, 727)
LSVT vs control* (*n* = 52)	1550	(944, 2217)
**Controlling for duration of illness**		
LSVT vs NHS SLT* (*n* = 48)	948	(436, 1502)
NHS SLT vs control* (*n* = 52)	307	(− 111, 748)
LSVT vs control* (*n* = 52)	1345	(825, 1923)
**Controlling for duration of illness and baseline VHI, EQ-5D-3L, and PDQ-39 communication scores**		
LSVT vs NHS SLT* (*n* = 44)	1105	(587, 1678)
NHS SLT vs control* (*n* = 47)	74	(− 341, 513)
LSVT vs control* (*n* = 47)	1175	(626, 1775)

^*^ denotes reference group

### Outcomes

VHI scores were similar between arms at baseline, but the PDQ-39 summary index was higher in the LSVT® LOUD arm (indicating poorer QoL) (Table 5). Consistent with this, the EQ-5D-3L score was lower in the LSVT® LOUD arm, and highest in the control arm; the reverse was the case for ICECAP-O although the scores are more similar between arms.

**Table 5:** Mean baseline outcomes by trial arm

**Outcome** Mean (95% CI)	**LSVT® LOUD** (*n* = 29)	**NHS SLT** (*n* = 30)	**Control** (*n* = 29)
VHI	41.7 (34.3, 49.4)	42.2 (33.3, 51.9)	42.4 (34.8, 50.9)
PDQ39-COMM	35.3 (27.3, 44.1)	32.8 (25.8, 40.8)	33.3 (27.0, 41.4)
PDQ-39 summary	32.4 (26.8, 37.8)	28.1 (23.6, 33.2)	26.4 (21.7, 31.6)
EQ-5D-3L	0.59 (0.48, 0.69)	0.64 (0.56, 0.72)	0.72 (0.66, 0.78)
ICECAP-O	0.79 (0.74, 0.84)	0.78 (0.70, 0.83)	0.75 (0.66, 0.82)

The VHI improved between baseline and 3 months for both the treatment arms, but this effect was only maintained at 6 and 12 months for patients in the LSVT® LOUD arm. The control arm showed worsening voice quality at 3 months, with improvements at 12 months, at which time point over half of participants had received SLT. Communication related QoL using the PDQ-39 communication domain also improved for both treatment arms at 3 months. Differences between arms in change in outcome measures at 3, 6, and 12 months, the end point for the economic analysis, are summarised in Table 6, adjusting for baseline values. There was no evidence of a difference in outcomes between arms at 12 months.

**Table 6:** Differences between treatment arms in voice, quality of life and QALYs at 3, 6, and 12 months, after adjusting for baseline values

**Outcome**	**LSVT® LOUD**^**a**^** vs NHS SLT** estimate (95% CI)	**NHS SLT**^**a**^** vs control** estimate (95% CI)	**LSVT® LOUD**^**a**^** vs control** estimate (95% CI)
**VHI**			
3 months 6 months 12 months	2.0 (− 7.0, 10.9) 8.4 (− 0.6, 17.4) 6.7 (− 3.7, 17.1)	6.7 (− 1.0, 14.4) 3.6 (− 3.6, 10.7) 0.03 (− 9.2, 9.3)	8.3 (− 0.9, 17.6) 12.1 (3.5, 20.8) 6.3 (v3.1, 15.6)
**PDQ39-COMM**			
3 months 6 months 12 months	3.6 (− 5.9, 13.2) − 0.06 (− 0.15, 0.04) 1.3 (− 10.4, 13.0)	4.5 (− 4.1, 13.7) 0.9 (− 8.2, 9.9) 1.4 (− 8.3, 11.1)	8.7 (− 1.1, 18.5) 6.6 (− 2.4, 15.6) 2.5 (− 9.2, 14.2)
**PDQ-39 Summary**			
3 months 6 months 12 months	1.4 (− 4.0, 6.7) 3.9 (− 2.2, 10.1) 1.1 (− 5.3, 7.0)	3.3 (− 1.0, 7.7) − 0.2 (− 5.1, 4.7) − 3.1 (− 7.6, 1.5)	5.2 (− 0.1, 10.4) 4.4 (− 0.7, 9.4) − 2.3 (− 8.8, 4.2)
**EQ-5D-3L**			
3 months 6 months 12 months	0.07 (− 0.03, 0.16) 0.05 (− 0.08, 0.17) 0.01 (− 0.14, 0.17)	− 0.15 (− 0.36, − 0.26) − 0.04 (− 0.15, 0.06) − 0.04 (− 0.17, 0.94)	− 0.09 (− 0.21, 0.04) − 0.00 (− 0.12, 0.12) − 0.02 (− 0.17, 0.14)
**ICECAP-O**			
3 months 6 months 12 months	− 0.01 (− 0.8, 0.06) − 0.05 (− 0.16, 0.05) − 0.06 (− 0.15, 0.04)	0.02 (− 0.05, 0.09) 0.08 (− 0.02, 0.17) 0.08 (− 0.01, 0.17)	0.01 (− 0.07, 0.09) 0.02 (− 0.11, 0.07) 0.01 (− 0.07, 0.09)
**Total QALYs**	0.03 (− 0.05, 0.10)	− 0.06 (− 0.15, 0.02)	− 0.04 (− 0.12, 0.05)

VHI ranges from 0 to 120; PDQ-39 summary index and communication domain score range from 0 to 100, where low score is good. Positive difference favours treatment

EQ-5D-3L ranges from − 0.59 to 1; ICECAP-O from 0 to 1, where high score is good. Negative difference favours treatment

^a^ reference arm

### Quality adjusted life years (QALYs)

QALYs were calculated over the 12 months, for the 74 patients with an EQ-5D-3L score at baseline, 3, 6, and 12 months. Absolute unadjusted QALYs were 0.56 for the LSVT® LOUD arm and 0.63 for both the NHS SLT and control arms (Table 3). However, there were only minimal differences in total QALYs between treatment arms, after adjusting for baseline EQ-5D-3L score (Table 6).

### Validity of outcome measures for PwPD

There was strong correlation between the voice measures, and between PD-related QoL (summary index and communication score) and voice outcomes (Table 7). EQ-5D-3L was strongly correlated to PDQ-39, and moderately correlated to the voice measures. The ICECAP-O measure was only weakly correlated with EQ-5D-3L and VHI. ICECAP-O was weakly correlated with voice measures. Negative correlations were due to the health economic outcomes scoring high for better outcomes and the PD-related scales scoring low for better outcomes.

**Table 7:** Convergence between baseline outcome measures (Pearson’s correlation coefficient)

	**PDQ-39**	**EQ-5D-3L**	**ICECAP-O**	**VHI**	**VR-QoL**	**PDQ_ COMM**
PDQ-39 Summary	1					
EQ-5D-3L	− 0.65	1				
ICECAP-O	− 0.24	0.20	1			
VHI	0.61	− 0.38	− 0.19	1		
VR-QoL	0.57	− 0.39	− 0.27	0.87	1	
PDQ_COMM	0.73	− 0.42	− 0.31	0.73	0.76	1

VHI ranges from 0 to 120 where low score is good; PDQ-39 summary index and communication domain score range from 0 to 100, where low score is good

EQ-5D-3L ranges from − 0.59 to 1; ICECAP-O from 0 to 1, where high score is good

Relationships between EQ-5D-3L and ICECAP-O dimensions were examined (Supplementary Table 7). Moderate correlations existed between ICECAP-O Control and EQ-5D-3L Mobility and Self-Care, suggesting similar constructs relating to independence and ability to do things without help. There were also moderate correlations between ICECAP-O enjoyment and EQ-5D-3L mobility; and between ICECAP-O Security and EQ-5D-3L Usual activities. The ICECAP-O attributes role and attachment had weak levels of correlation with the EQ-5D-3L dimensions, reflecting the different concepts they are measuring.

The relationship between the dimensions of PDQ-39 and the economic measures EQ-5D-3L and ICECAP-O were explored (Supplementary Tables 8 and 9). PDQ-39 and EQ-5D-3L dimensions relating to similar constructs are strongly correlated, for example ADL and self-care. ICECAP-O and PDQ-39 dimensions were generally more weakly correlated.

The group comparisons (Table 8) assessed whether the outcome measures are able to distinguish between known patient groups based on each of the two variables (severity, and patient perception of speech impairment). Participants with more severe PD had significantly lower EQ-5D-3L scores, and worse PDQ-39 summary and communication scores, but there was no evidence of a difference in ICECAP-O, although a weak relationship was seen with VHI. Participants who reported that speech impacted on their social activities had much higher (worse) scores on VHI and PDQ-39 summary index and communication domain score, but there was no evidence of a difference in EQ-5D-3L score or ICECAP-O score.

**Table 8:** Group comparisons: sensitivity of outcome measures to differences between patient groups at baseline

	**Severity**^a^ Difference (95% CI)	**Speech impacts on social activities**^b^ Difference (95% CI)
VHI	8.8 (− 1.7, 19.4)	28 (17, 38)*
PDQ-39 Summary	10.1 (3.7, 16.6)*	14.9 (7.8, 21.9)*
PDQ_COMM	14.1 (4.7, 23.6)*	21.7 (10.9, 32.4)*
EQ-5D-3L	− 0.20 (− 0.31, − 0.10)*	− 0.11 (− 0.25, 0.04)
ICECAP-O	− 0.01 (− 0.1, 0.08)	− 0.02 (− 0.11, 0.07)

Voice Handicap Index (VHI) ranges from 0 to 120; PDQ-39 summary index and communication domain score range from 0 to 100, where low score is good

EQ-5D-3L ranges from -0.59 to 1; ICECAP-O from 0 to 1, where high score is good

^a^Reference group: H&Y 2.5 and above

^b^Reference group: yes response

^*^p < 0.01

## Discussion

### Findings

This was an exploratory economic evaluation alongside a pilot RCT comparing costs and outcomes of LSVT® LOUD, NHS SLT, and no treatment at 12 months. The LSVT® LOUD arm received more intensive SLT over a longer period than the NHS SLT arm. Between 6 and 12 months, 18 patients in the control arm received SLT, and over the 12 months received only a slightly lower number of sessions that the NHS SLT arm. There was no evidence of a difference in outcomes at 12 months, although some evidence of improvement in voice outcomes at 3 months. NHS costs were £3288 for the LSVT® LOUD arm, £2033 for the NHS SLT arm and £1738 for the control arm. Differences in cost were largely accounted for by differences in SLT costs. Other costs were similar between trial arms and comparable to those reported in other studies with patients of similar severity of PD [31, 36].

Regarding validity of outcome measures, EQ-5D-3L was more strongly related to voice impairment than ICECAP-O, and was sensitive to differences in voice impairment in patients. This could be because voice impairment is related to overall PD severity and other PD related impairment, which is captured well by EQ-5D-3L. EQ-5D-3L and PDQ39 captured similar health constructs in four dimensions: mobility, pain/discomfort, anxiety/emotional wellbeing, and self-care/ADL. The instruments showed strong correlations for these dimensions, and both instruments also showed worse scores for patients with more severe PD [37]. ICECAP-O was more weakly correlated with PDQ-39 than EQ-5D-3L, and ICECAP-O attributes were correlated with the PDQ-39 dimensions Stigma, Communication and Emotional wellbeing. This suggests that ICECAP-O is aligned to different constructs to EQ-5D-3L [38], and the measures are complementary.

### Strengths

The PD COMM Pilot is the first study to examine the economic aspects of speech and voice difficulties in PD, a difficult area for research, given that speech is one of a wide range of health aspects affected by PD, for which it is not clear which aspects of costs should be considered. The resource use questionnaire captured NHS costs in detail, allowing a detailed analysis of resource use as part of a cost consequence analysis. Collection of information on SLT visits directly from patients was valuable as it quantified the SLT received by the control arm. Care was taken to ensure that SLT was not double counted, by screening the patient completed resource questionnaire to remove SLT related to the trial. Further, the study includes a wide range of outcomes, relating to speech, PD, HRQoL, and capability, allowing analysis of the validity of outcome measures for SLT in PD, an area where there is currently a gap in research evidence.

### Limitations

In this pragmatic trial, patients in the control arm received an average of 5 SLT sessions over the 12 months of the study, thus diluting the comparison between the LSVT® LOUD and NHS SLT arms. This impacted on the economic evaluation, which has a 12-month time horizon. This has been addressed in the main trial protocol [39] for which participants in the control arm have SLT deferred for at least 12 months, unless deemed clinically necessary.

This analysis was undertaken using data from a pilot study, and as such, the results are preliminary as they are based on a sample size not powered to detect difference in cost-consequence analysis, and using data collection methods which were being tested. There were differences between the participants in the study at baseline in duration of disease, and in PDQ39 and EQ-5D-3L scores. While response rates overall were high, the cumulative impact of missing data meant that complete cases were available for analysis of resource use data for 85% of cases. Outcomes data was less complete, particularly for the voice measures. Multiple imputation was not undertaken, in line with the main analysis of the pilot study [14]. Given the insufficient sample size for a cost-consequence analysis and missing data, findings from the analysis should be treated with caution.

There were some limitations in the data available for costing NHS services. Coding of the free text responses was needed to categorise outpatient activity, and also resource use reported under “other” by patients. Resource use in the last follow-up period may have been under reported by patients, as resources used were similar for this 6-month period to the earlier 3-month periods. Although bootstrapping of costs was undertaken to allow for skewed data, there was one notable outlier on resource use in the LSVT® LOUD arm for which sensitivity analysis was undertaken. A full assessment of NHS costs was not undertaken, as inpatient costs and non-PD-related medication costs were not captured. The resource use questionnaire did not enable patients to report which social care costs were self-funded. Given the shifting landscape of social care costs [40], this is a limitation.

Regarding the analysis of outcomes, it should also be noted that the distribution of ICECAP-O and EQ-5D-3L scores at baseline was skewed, and so some caution is needed in interpreting the correlation between scores. Furthermore, Spearman’s rank correlation was used to compare dimension scores between outcome measures; however, the method assumes both measures are continuous. This is not the case for measures such as the EQ-5D-3L and ICECAP-O which are categorical with three and four levels respectively.

### Implications for economic analysis of full trial

Based on the preliminary analysis of outcome measures, the most appropriate economic measure appears to be EQ-5D-3L, as this was sensitive to differences in voice impairment. ICECAP-O is recommended as an additional economic measure, and analysis of the full trial data will allow the opportunity to compare ICECAP-O and EQ-5D-3L longitudinally, as recommended by Mitchell [41]. It would be informative to analyse the relationship of the individual attributes of ICECAP-O with voice measures and to compare patient reported and clinically assessed voice measures, in relation to severity of PD, as the relationship between patient’s perception and clinical measures is unclear [3]. Further, given that the LSVT® LOUD is an intensive programme for which burden of treatment is an important consideration [42], it would be interesting to further understand the relationship between severity and participation in treatment: PD patients have a wide range of symptoms and impairments and there may be trade-offs in which symptoms are a priority to address from the patient’s perspective. A wider cost–benefit approach could be considered for this [43], taking account of patient and carer time in undertaking treatment, as well as NHS costs, and also carer outcomes [44].

## Conclusions

The cost consequence analysis provides a detailed analysis of costs of PD for a community sample, and established the costs of LSVT® LOUD and NHS SLT. EQ-5D-3L was more strongly related to voice impairment than ICECAP-O and was sensitive to differences in voice impairment in patients. Hence, the preferred economic outcome measure for the full trial is EQ-5D-3L, although it is recommended that ICECAP-O is also included, as there is evidence that this captures some important dimensions of PD related QoL which are not included in EQ-5D-3L.

The full trial will enable a more conclusive assessment of the effectiveness and cost-effectiveness of SLT for PwPD from an NHS perspective. Alternative perspectives for economic evaluation could also be considered, for example considering carer outcomes and patient costs. Further, the full trial will also provide the opportunity to assess with a larger sample which economic measure are sensitive to communication and voice impairment, the relationship with severity of PD, and the contribution of communication factors to overall QoL.

## Supplementary Information

**Additional file 1: Table S1**. Speech and language therapy (SLT) set-up costs. **Table S2**. Derivation of unit costs: sources and assumptions. **Table S3**. Resource use per patient over 12 months (NHS and social care funded). **Table S4**. Mean medication costs by drug type over 12 months, per patient (2014/15 costs). **Table S5**. Resource use per patient over 12 months (privately funded). **Table S6**. Patient funded care costs and out of pocket expenses over 12 months, per patient. **Table S7**. Convergence between index scores of EQ-5D-3L and ICECAP-O dimensions (Spearman’s rank correlation). **Table S8**. Convergence between index scores of PDQ39 dimensions and ICECAP-O responses (Spearman’s rank correlation). **Table S9**. Convergence between index scores of PDQ39 dimensions and EQ-5D-3L responses (Spearman’s rank correlation)

## Data Availability

The dataset analysed during the current study is available from the corresponding author on reasonable request.

## Abbreviations

EQ-5D-3L: EuroQoL 5 Dimensions-3 levels
ICECAP-O: ICECAP capability measure for older people
H&Y: Hoehn and Yahr severity score
LSVT® LOUD: Lee Silverman Voice Treatment
NHS: National Health Service
PD: Parkinson’s disease
PwPD: People with Parkinson’s disease
PDQ-39: Parkinson’s Disease Questionnaire-39
PDQ COMM: Parkinson’s Disease Questionnaire-39, communications domain
SLT: Speech and language therapy
VHI: Voice Handicap Index
