# Supplementary material for: Lee Silverman Voice Treatment versus standard speech and language therapy versus control in Parkinson’s disease: preliminary cost-consequence analysis of the PD COMM pilot randomised controlled trial
Source: Pilot Feasibility Stud. 2021 Aug 9;7:154. doi: 10.1186/s40814-021-00888-y (PMC8351093; doi:10.1186/s40814-021-00888-y)
Supplement: Supplementary file 1 — Additional file 1: Table S1. Speech and language therapy (SLT) set-up costs. Table S2. Derivation of unit costs: sources and assumptions. Table S3. Resource use per patient over 12 months (NHS and social care funded). Table S4. Mean medication costs by drug type over 12 months, per patient (2014/15 costs). Table S5. Resource use per patient over 12 months (privately funded). Table S6. Patient funded care costs and out of pocket expenses over 12 months, per patient. Table S7. Convergence between index scores of EQ-5D-3L and ICECAP-O dimensions (Spearman’s rank correlation). Table S8. Convergence between index scores of PDQ39 dimensions and ICECAP-O responses (Spearman’s rank correlation). Table S9. Convergence between index scores of PDQ39 dimensions and EQ-5D-3L responses (Spearman’s rank correlation) [file 40814_2021_888_MOESM1_ESM.docx]

**Supplementary tables**

**Table S1: Speech and language therapy (SLT) set-up costs**

| **Speech and language therapy (SLT) services** | |  |
| --- | --- | --- |
| Average PwPD on SLT service caseload per year | 13 | Estimated from 63 services with a combined caseload of 820 PwPD |
| Number of trained LSVT SLTs | 2 | Most services were staffed with 1–3 full time equivalent therapists seeing PwPD |
| Cost for training | £1,210 | PD Comm study team estimate training at £505/person, plus estimate of £100/person travel and subsistence |
| **Cost for training per PwPD** | **£18.60** | Assumes training repeated every 5 years |

Source: Parkinson’s UK, 2016

**Table S2: Derivation of unit costs: sources and assumptions**

| **Type of resource** | **Resource** | **PSSRU** ^1^ | | **PD comm units and costs applied** | | | **Notes and assumptions** |
| --- | --- | --- | --- | --- | --- | --- | --- |
|  |  | **Unit** | **NHS Unit cost £** | **Unit** | **NHS Unit cost £** | **Private Unit cost £** |  |
| SLT treatment | Therapist time | /session | 52 | /session | 65 |  | Est 75 min staff time/session to cover set-up, notes etc , travel costed at hourly rate |
| Primary care and community nursing services | GP home visits | /visit | 68 | /visit | 68 |  | Cost/minute - £2.90; 11.4 minutes for visit plus 12 minutes for travel time. |
|  | GP surgery visits | /visit | 33 | /visit | 33 |  | Based on GP pay costs, not full practice costs |
|  | Practice nurse visits | /face-face contact hour | 56 | /visit | 14 |  | Average consultation time 15.5 minutes |
|  | Practice nurse home visits | /face-face contact hour | 56 | /visit | 28 |  | Home visit length assumed to be longer to allow for travel time |
|  | Parkinson’s disease nurse specialist visits | /working patient related hour | 65 | /visit | 65 |  | Assumes community based nurse specialist. Assume visit is 1 hour. |
|  | Health visitor visits | /working patient related hour | 66 | /visit | 33 |  | Assume length consultation as for practice nurse |
| Therapists and other healthcare professionals | Social worker visits | /client related hour | 79 | /visit | 79 |  | Adult social care. Based on 1 hour contact (research team advice) |
|  | Physiotherapist visits | /attendance | 34 | /visit | 34 | 50 | NHS: Hospital based: mean cost/follow-up attendance |
|  | Occupational therapist visits | /attendance | 53 | /visit | 53 | 50 | NHS: Hospital based: mean cost/follow-up attendance |
|  | SLT visits | /hour | 52 | /visit | 65 | 50 | NHS: Based on Band 7 Advanced SLT (Band 6 equivalent is £44 per working hour); similar to mean cost/attendance in hospital (£67) |
|  | Other private practitioner |  |  | /visit |  | 50 | Private costs estimated at £50/session based on costs quoted on private provider websites^2^. |
|  | Other |  | 46 | /visit | 51 | 50 | Estimated as average of other therapy costs |
| Outpatient appointments | PD consultant appointments | /appointment | 118 | /appointment | 118 |  |  |
|  | PD other appointments | /appointment | 91 | /appointment | 91 |  |  |
|  | Other appointments | /appointment | 118 | /appointment | 118 |  |  |
| Social care services | Home care/help | /hour | 24 | /visit | 12 |  | Approx 30 min per visit |
|  | Meals on wheels | /attendance | NA | /meal | 4 |  | Source: National Association for Care Catering^3^. |
|  | Day centre | /attendance | 57 | /day | 57 |  | Assumes a day is one attendance. |
|  | Luncheon club | /attendance | NA | /meal | 4 |  | Assumes same cost/meal as for meals on wheels |
|  | Respite service* | /hour | 24 | /hour | 48 |  | Assumes costs as home care/ help per hour but for 2 hours/visit |
| Residential care | Residential care | /week | 1110 | /week | 1110 | 595 |  |

Sources: 1 - Curtis and Burns, 2015, 2 - Nuffield Health, 2016, 3 - National Association for Care Catering

*A respite service provides an individual to go to the home of a patient allowing carers free time away from caring duties.

**Table S3: Resource use per patient over 12 months (NHS and social care funded)**

| **Type of resource**  Mean (SD) | **LSVT**  (n=24) | **NHS SLT**  (n=24 ) | **Deferred**  (n=28) |
| --- | --- | --- | --- |
| **Primary care and community nursing visits** | |  |  |
| GP surgery | 5.0 (5.2) | 5.0 (4.1) | 4.3 (4.3) |
| GP home | 0.83 (3.5) | 1.0 (2.4) | 0.25 (0.7) |
| Practice nurse | 2.0 (1.9) | 1.5 (2.1) | 1.5 (2.1) |
| Practice nurse home | 0.46 (1.4) | 0.5 (1.4) | 0.36 (1.5) |
| Parkinson’s disease nurse specialist | 1.6 (1.3) | 1.1 (1.2) | 1.7 (0.9) |
| Health visitor | 0.46 (1) | 0.13 (0.45) | 0.11 (0.42) |
| **Therapists and other healthcare professional visits** | |  |  |
| Social worker | 0.21 (0.66) | 0.13 (0.45) | 0 (0) |
| Physiotherapist | 2.5 (4.2) | 2.5 (5) | 1.8 (2.4) |
| Occupational therapist | 0.58 (1.2) | 0.42 (1.2) | 0.25 (0.7) |
| Other | 5.5 (25.7) | 1 (2.9) | 0.32 (1.5) |
| **Outpatient appointments** |  |  |  |
| Parkinson’s disease consultant | 2.3 (2) | 1.8 (1.9) | 1.1 (1.2) |
| Parkinson’s disease other | 0.25 (0.74) | 0.29 (0.75) | 0.64 (1.4) |
| Other | 2.2 (2.1) | 2.6 (2.6) | 2.3 (3.2) |
| **Social care services, per week** |  |  |  |
| Home care/help visits | 0.29 (1.4) | 1.6 (6.3) | 0.04 (0.19) |
| Day centre | 0.04 (0.2) | 1 (5.1) | 0 (0) |
| Luncheon club meals | 0.04 (0.2) | 0 (0) | 0 (0) |
| Respite service, days | 0.08 (0.41) | 1 (4.9) | 0 (0) |
| Other | 1.4 (6.3) | 0 (0) | 0 (0) |
| Residential care, n (%) | 0 (0) | 2 (8) | 0 (0) |

A small number of patients visited privately funded services (Table 6), notably physiotherapy, with dentists, chiropractors and chiropodists being mentioned by patients under “other” privately funded services.

**Table S4: Mean medication costs by drug type over 12 months, per patient (2014/15 costs)**

| **Type of resource**  Mean (SD) | **LSVT® LOUD**  (n=24)  £ | **NHS SLT**  (n=24)  £ | **Control**  (n= 28)  £ |
| --- | --- | --- | --- |
| **Medication type** |  |  |  |
| Dopamine agonist | 585.18 (714.24) | 345.67 (595.80) | 278.54 (521.60) |
| Levodopa | 59.22 (57.63) | 71.87 (77.00) | 48.00 (63.65) |
| MAO-B inhibitors | 99.36 (231.32) | 137.38 (257. 15) | 152.47 (287.75) |
| COMT inhibitors | 22.78 (106.69) | 10.89 (53.35) | 11.67 (61.74) |
| Stalevo (combination drug) | 116.99 (193.76) | 50.59 (115.55) | 86.72 (158.25) |
| **Total medication costs** | **883 (691)** | **616 (646)** | **577 (598)** |

**Table S5: Resource use per patient over 12 months (privately funded)**

| **Type of resource**  Mean (SD) visits | **LSVT**  (n=24) | **NHS SLT**  (n=24) | **Deferred**  (n=28) |
| --- | --- | --- | --- |
| Physiotherapist | 0.38 (1.2) | 0.21 (0.83) | 0.43 (1.6) |
| Occupational therapist | 0 (0) | 0 (0) | 0.04 (0.19) |
| SLT | 0 (0) | 0 (0) | 0.29 (1.5) |
| Other private - practitioner | 0.50 (1.8) | 1.40 (5.3) | 0.93 (3.8) |
| Other private | 0.71 (1.9) | 0.92 (2.2) | 0.46 (2.3) |

**Table S6: Patient funded care costs and out of pocket expenses over 12 months, per patient**

| **Type of resource**  Mean (SD) | **LSVT**  **(n=24)**  **£** | **NHS SLT**  **(n=24)**  **£** | **Deferred**  **(n=28)**  **£** |
| --- | --- | --- | --- |
| **Therapists/other healthcare professionals** | 79 (156) | 127 (361) | 93 (288) |
| **Out of pocket expenses** |  |  |  |
| Travel | 75 (156) | 40 (51) | 86 (138) |
| Parkinson’s medication | 2 (9) | 6 (21) | 31 (110) |
| Any other costs | 267 (829) | 233 (602) | 8 (33) |
| **All patient costs** | 424 (851) | 405 (750) | 218 (337) |

**Table S7: Convergence between index scores of EQ-5D-3L and ICECAP-O dimensions (Spearman’s rank correlation)**

|  | **EQ-5D-3L** | | | | |
| --- | --- | --- | --- | --- | --- |
| **ICECAP-O** | **Anxiety/ depression** | **Mobility** | **Pain/ discomfort** | **Self-care** | **Usual activities** |
| Attachment | 0.09 | -0.26 | -0.11 | -0.05 | -0.15 |
| Security | -0.27 | -0.12 | -0.21 | -0.04 | -0.32 |
| Role | -0.06 | -0.25 | -0.10 | -0.22 | -0.27 |
| Enjoyment | -0.16 | -0.31 | -0.13 | -0.19 | -0.29 |
| Control | -0.09 | -0.40 | -0.19 | -0.37 | -0.26 |

**Table S8: Convergence between index scores of PDQ39 dimensions and ICECAP-O responses (Spearman’s rank correlation)**

|  | **ICECAP-O** | | | | |
| --- | --- | --- | --- | --- | --- |
| **PDQ39** | **Attachment** | **Security** | **Role** | **Enjoyment** | **Control** |
| Activities of Daily Living | -0.03 | -0.05 | -0.27 | -0.21 | -0.42 |
| Bodily discomfort | -0.15 | -0.03 | -0.17 | -0.28 | -0.30 |
| Cognition | -0.03 | -0.08 | -0.21 | -0.19 | -0.28 |
| Communication | -0.15 | -0.20 | -0.38 | -0.28 | -0.41 |
| Emotional | -0.10 | -0.45 | -0.37 | -0.38 | -0.14 |
| Stigma | -0.21 | -0.29 | -0.34 | -0.33 | -0.18 |
| Support | -0.32 | -0.22 | -0.21 | -0.28 | -0.28 |
| Mobility | -0.17 | -0.16 | -0.42 | -0.36 | -0.47 |

**Table S9: Convergence between index scores of PDQ39 dimensions and EQ-5D-3L responses (Spearman’s rank correlation)**

|  | **EQ-5D-3L** | | | | |
| --- | --- | --- | --- | --- | --- |
| **PDQ39** | **Anxiety/ depression** | **Mobility** | **Pain/ discomfort** | **Self-care** | **Usual activities** |
| Activities Daily Living | 0.25 | 0.48 | 0.28 | 0.72 | 0.44 |
| Bodily discomfort | 0.26 | 0.41 | 0.50 | 0.35 | 0.45 |
| Cognition | 0.25 | 0.26 | 0.26 | 0.43 | 0.31 |
| Communication | 0.31 | 0.31 | 0.13 | 0.39 | 0.28 |
| Emotional | 0.56 | 0.22 | 0.35 | 0.35 | 0.43 |
| Stigma | 0.15 | 0.27 | 0.09 | 0.21 | 0.28 |
| Support | 0.26 | 0.28 | 0.22 | 0.39 | 0.23 |
| Mobility | 0.34 | 0.66 | 0.23 | 0.50 | 0.45 |
